# SOCIAL DETERMINANTS OF HEALTH COMMUNITY-LEVEL SOCIAL DETERMINANTS OF MORTALITY IN OLDER ADULTS: AN ASSESSMENT OF RHODE ISLAND CITIES AND TOWNS

**DOI:** 10.1093/geroni/igz038.2882

**Published:** 2019-11-08

**Authors:** Steven A Cohen, Julia Broccoli, Heather O’Neill, Mary L Greaney

**Affiliations:** 1 University of Rhode Island, Kingston, Rhode Island, United States; 2 PixelsforHumans, East Greenwich, Rhode Island, United States

## Abstract

Addressing the causes of place-based health disparities among older adults have focused on understanding social determinants of health on a large geographic level, such as region, state, or county. However, there is a growing realization for the need to understand how place-based characteristics at smaller geographic areas relate to population health and contribute to successful aging. The purpose of this study was to assess the magnitude of the associations between place-based social determinants and life expectancy (LE) among older adults and related measures. Methods: LE at age 50 (LE50) and age 65 (LE65), and the age-specific mortality rate (ASMR) for ages 65+ (ASMR65) were calculated from mortality data (2009-2011) from the Rhode Island (RI) Department of Health (RIDoH) using abridged life table methods for each RI city/town. City/town-specific LE and ASMR were linked to the US Census, RIDoH, and other databases that include social determinants: demographics, household composition, wealth, education, environment, food insecurity, crime, transportation, and rural-urban status. Bivariate and partial correlations were examined between city/town-level LE50, LE65, and ASMR65. Results: LE50, (range: 29.3-34.0 years) was most strongly associated with the percent of the population with at least a bachelor’s degree (r=0.652, p<0.001), per capita income (r=-0.632, p < 0.001), and percent multigenerational households (r=-0.489, p=0.003). The associations between both LE65 and ASMR65 and examined social determinants were more attenuated, however. Discussion: These results highlight substantial place-based disparities in mortality and potentially addressable social determinants that could improve population health for older adults and reduce place-based disparities among neighboring communities.

